# Toward reliable calcification detection: calibration of uncertainty in object detection from coronary optical coherence tomography images

**DOI:** 10.1117/1.JBO.28.3.036008

**Published:** 2023-03-27

**Authors:** Hongshan Liu, Xueshen Li, Abdul Latif Bamba, Xiaoyu Song, Brigitta C. Brott, Silvio H. Litovsky, Yu Gan

**Affiliations:** a Stevens Institute of Technology, Biomedical Engineering Department, Hoboken, New Jersey, United States; b Columbia University, Department of Electrical Engineering, New York, United States; c Icahn School of Medicine at Mount Sinai, New York, United States; d University of Alabama at Birmingham, School of Medicine, Birmingham, Alabama, United States

**Keywords:** optical coherence tomography, coronary artery disease, deep learning, calibration

## Abstract

**Significance:**

Optical coherence tomography (OCT) has become increasingly essential in assisting the treatment of coronary artery disease (CAD). However, unidentified calcified regions within a narrowed artery could impair the outcome of the treatment. Fast and objective identification is paramount to automatically procuring accurate readings on calcifications within the artery.

**Aim:**

We aim to rapidly identify calcification in coronary OCT images using a bounding box and reduce the prediction bias in automated prediction models.

**Approach:**

We first adopt a deep learning-based object detection model to rapidly draw the calcified region from coronary OCT images using a bounding box. We measure the uncertainty of predictions based on the expected calibration errors, thus assessing the certainty level of detection results. To calibrate confidence scores of predictions, we implement dependent logistic calibration using each detection result’s confidence and center coordinates.

**Results:**

We implemented an object detection module to draw the boundary of the calcified region at a rate of 140 frames per second. With the calibrated confidence score of each prediction, we lower the uncertainty of predictions in calcification detection and eliminate the estimation bias from various object detection methods. The calibrated confidence of prediction results in a confidence error of ∼0.13, suggesting that the confidence calibration on calcification detection could provide a more trustworthy result.

**Conclusions:**

Given the rapid detection and effective calibration of the proposed work, we expect that it can assist in clinical evaluation of treating the CAD during the imaging-guided procedure.

## 1 Introduction

Optical coherence tomography (OCT) can acquire high-resolution cross-sectional images of coronary arteries. The high-quality and detailed information from coronary OCT images facilitates the treatment of coronary artery disease (CAD). CAD causes 1 of every 5 deaths in Europe^1^ and 1 of every 6 deaths in the United States,^2^ and it remains one of the leading causes of morbidity and mortality in developed countries.^3^ Coronary atherosclerosis is caused by the gradual buildup of plaque resulting from the depositing of calcium, lipids, and macrophages from the luminal blood into the arterial intima. Coronary atherosclerosis compounds and augments the risks of heart attack and heart failure. When treated improperly or left unattended, coronary atherosclerosis blocks the pathways to the heart’s main arteries, known as the coronary arteries. The potential effects of plaque in CAD include chest pain, shortness of breath, heart failure, myocardial infarction, and sudden death.

A typical treatment for CAD is percutaneous coronary intervention (PCI), which is a nonsurgical procedure used to treat the narrowing of the heart’s coronary arteries. Unidentified calcified tissues within a narrowing artery often negatively impact the benefits of treatment. Approximately 700,000 PCIs are performed every year in the United States, and calcifications have been found in 17% to 35% of patients undergoing the procedure,^4^^–^^6^ highlighting a need to precisely locate the existence and extent of calcifications. Most PCI procedures involve using stents to open up obstructed coronary arteries.^7^ During the PCI procedure, a catheter with a tiny, folded balloon on its tip is inserted into the blood vessels until it arrives at the site where the plaque buildup is causing a blockage. At that point, the balloon is inflated to compress the plaque against the artery walls, therefore widening the passageway and restoring blood flow to the heart. After that, the balloon is deflated and removed. A stent implantation is performed in the plaque buildup area to keep the artery open after removing the balloon.^8^ Excess coronary calcification is highly related to the suboptimal deployment of the stent in the coronary during the PCI.^9^ Major calcifications are of great concern for two reasons.^10^ Calcifications can lead to stent underexpansion and strut malapposition. Malapposition of stent struts (e.g., an empty space between the strut and the adjacent vessel wall) might preclude healthy endothelial tissue growth. Even though stent deployment is generally effective in the short term, stent efficacy can be reduced and the risk can be increased by adverse clinical events, such as in-stent restenosis and thrombosis in the medium- and long-term.^11^^–^^17^

Coronary imaging guidance during PCI is one of the key determinants of treatment outcomes. Imaging is integral to every stage of PCI, such as assessment of lesion severity, preprocedural planning, optimization, and management of immediate complications.^18^^,^^19^ OCT has significant advantages for characterizing coronary calcification that typically has a signal-poor area with sharply delineated borders.^20^ A typical OCT system can achieve a high axial resolution at the micron level and a penetration depth of up to 2 mm, indicating superior imaging capability.^21^^,^^22^ The detection of calcified regions within coronary OCT images is critical for intervention.^23^ On account of this, developing an object detection algorithm that is capable of detecting calcification in OCT images is essential.

Deep learning has been increasingly explored in analyzing the diseased tissue in coronary OCT images.^24^ In existing research works,^25^^–^^30^ extensive studies have been conducted to automatically identify plaque in coronary OCT images. A weighted majority voting from different convolutional neural networks (CNN)^26^ was used to solve the multiclass classification problem of pathological formations in coronary artery tissues. A deep convolutional architecture named SegNet segmented calcification in coronary OCT images.^10^ A two-step deep learning approach^27^ characterized plaques in coronary arteries in OCT images by first localizing the major calcification lesions using the CNN model and then applying the deep learning model (SegNet) to provide pixel-wise classifications of calcified plaques. A modified deep convolutional segmentation model UNet^28^ was used to identify calcification in coronary OCT images. The segmentation module in MASK-RCNN was employed to identify the erosion region.^31^ Currently, the most popular way to perform automated analysis on OCT images is deep learning-based segmentation, which makes the pixel-wise classification and outputs the detailed shape and location of the tissue of interest. Demonstrably, the segmentation architecture results in large computational costs due to the burden of pixel-wise classification. By virtue of this, a more efficient way of enacting automated analysis of coronary images is through the use of object detection, which outputs the bounding box of the tissue region rather than the pixel-wise classification of the tissue region, to efficiently identify the diseased region in coronary images.

Although existing works also focus more on increasing the accuracy of deep learning models, the quality of predictions can be negatively impacted by overconfident deep learning models.^32^ The problem of overconfidence can be produced by deep learning models in the form of providing high confidence scores for predictions.^33^^–^^35^ In general, recalibration methods of the well-trained model, such as Platt scaling,^36^ histogram binning,^37^ and temperature scaling,^38^ can improve the calibration of the overconfident prediction results. In addition, model ensemble methods^39^^,^^40^ can also reduce overconfidence by aggregating the prediction results over multiple models. However, there are limited studies on correcting overconfident predictions in coronary OCT images. In OCT-related CAD treatment, overconfidence could be dangerous as confidence is often learned as the likelihood that the prediction is correct. Therefore, in safety-critical and risk-sensitive applications in clinical diagnosis, it is crucial to quantify and calibrate the uncertainty of predictions.

In this work, we aim to achieve reliable calcification detection for patients with CAD to boost the efficiency of clinical diagnosis. We summarize our contributions as follows.

1. We detect calcification in coronary OCT images via a deep learning-based object detection model. The object detection process delineates the bounding box of the calcified region within OCT images, providing a computationally efficient solution in comparison with conventional segmentation methods.
2. We propose calibrating the confidence of the coronary object detection task. We use a dependent logistic calibration method to reduce the bias in the prediction uncertainty.
3. We quantitatively and qualitatively evaluate the effectiveness of the proposed work on a human coronary dataset. The experimental results demonstrate the accuracy and speed of calcification detection and the effectiveness at reducing the bias of confidence among the three most popular object detection methods.

## 2 Methods

**Fig. 1:**
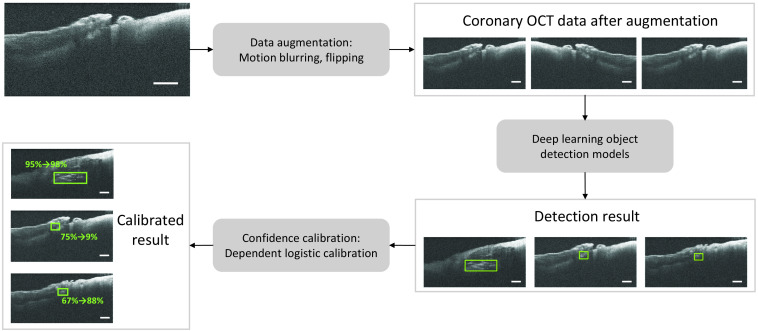
Flowchart of the proposed work. Scale bar: 500  μm.

The workflow is shown in Fig. 1. The steps are as follows: (1) the coronary OCT data are first processed by a data augmentation module to create motion-blurred and horizontally flipped copies of each original OCT image. (2) The coronary OCT data after augmentation are trained by deep learning object detection models, and the detection results on test data are output. (3) Detections containing bounding box coordinates and confidence scores are processed through dependent logistic calibration, and a calibrated confidence score is output for each predicted bounding box.

### 2.1 Data Collection

Samples are imaged by the spectral domain OCT system (Thorlabs Ganymede, Newton, New Jersey, United States) with an axial resolution of 3  μm and a lateral resolution of 4  μm in air. Autopsy specimens of human heart vessels are collected and imaged through the same protocol given in Refs. 41 and 42. All images are acquired in the laboratory at the University of Alabama.

### 2.2 Data Augmentation

Various data augmentation techniques have been proposed to improve the performance of deep learning models.^43^ During imaging, the quality of OCT images may be impacted due to degradation caused by motion blur,^44^^,^^45^ which can be caused by sample and device movement.^46^^–^^48^ A motion blur filter is used to simulate this effect of real-world conditions.^49^ Other common augmentation strategies, such as flipping, cropping, scaling, Gaussian noise, rotation, and shears, are routinely performed.^50^ Noticeably, we do not prefer vertical flipping or rotation in OCT images because the light propagates in a fixed direction, and applying such methods will change the nature of OCT images. Therefore, in this work, two copies of each OCT image are created by applying a motion-blurring filter and flipping horizontally for training the deep learning model.

### 2.3 Object Detection

**Fig. 2:**
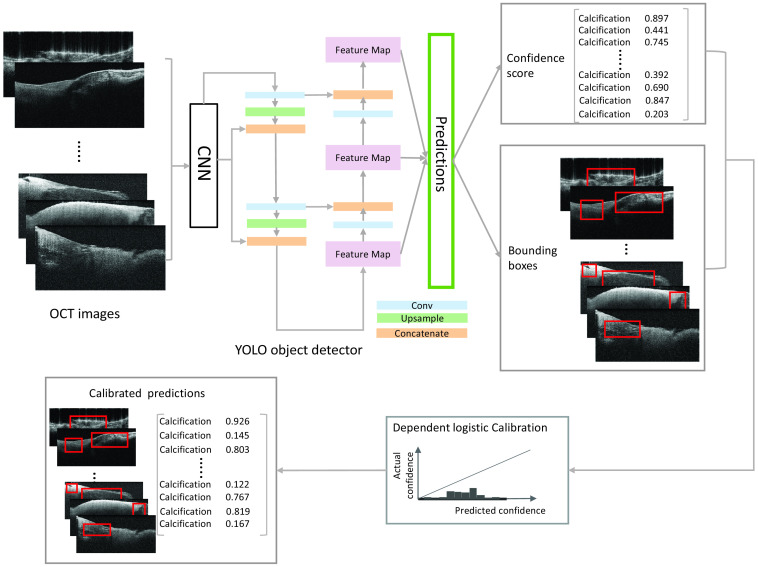
Schematic of YOLO object detector and calibration.

Object detection creates bounding box regions that identify an object’s position, size, and class within an image. We opt to use You-Only-Look-Once v5 (YOLO)^51^ to rapidly identify the bounding box and tissue types within an OCT image. Because of its lightweight and feature-reuse properties, the YOLO architecture is powerful at realizing fast and accurate detection. As the conceptual schematic shown in Fig. 2, to better predict objects of different sizes, YOLO enhances the detection performance by utilizing different scales of feature maps that are generated by applying filters to the input image or the feature map output of the prior layers.

The predictions have the outputs in two branches: confidence scores (confidence) and bounding boxes ($x_{\text{center}}$, $y_{\text{center}}$, width, and height). In the confidence score branch, the confidence score indicates a certain level that the prediction is true. In the bounding boxes branch, the values of the center coordinate, together with the width and height of the bounding box, depict the location of the predicted bounding box.

### 2.4 Uncertainty Measurement and Confidence Calibration

The common use of calibration is for the classification task, in which only the confidence score is utilized for a given image. In object detection, one additional piece of information that can be included for calibration is the location and scale of the bounding box. Therefore, in the object detection task, the criterion of a calibrated model is defined as the precision of a prediction given the confidence, class category, and bounding box information,^52^ as in the following equation:

P(z=1|p=^conf,y=^y,r=^r)=conf,∀  conf∈[0,1],  y∈Y,  r∈[0,1]K, (1)

where z=1 indicates that the prediction is correct, conf denotes the confidence of prediction, y is the predicted class in the set of all classes denoted by Y, and r is the bounding box information with k dimensions.

The expected calibration error (ECE) is used to measure the uncertainty of the prediction of the deep learning model. The ECE of object detection is calculated by binning the confidence $\hat{p}$ into M equally spaced bins. Samples with different confidence scores fall into corresponding bin m. $B_{m}$ is the number of samples in a bin, and N is the number of total samples. The Prec is the precision that represents the correct predictions among all predictions as defined in Eq. (4), and the conf denotes the average confidence score of the predictions. The ECE is given by

$$\mathrm{ECE}=\overset{M}{\underset{m=1}{\sum }}\frac{\left|B_{m}\right|}{N}|\mathrm{Prec}(m)-\mathrm{conf}(m)|.$$ (2)

For confidence calibration, in this work, we take two additional bounding box pieces of information, the center-x and center-y positions, along with the confidence score to calibrate the prediction results using the dependent logistic calibration,^52^ with the multivariate probability density function being used to model the log-likelihood ratio (lr) of the combined input(confidence,bounding box). Taking the correlations between the confidence and bounding box into consideration, the calibration map is defined as g and is given as

$$g(\text{input})\approx \frac{1}{1+e^{-\mathrm{lr}(\text{input})}},\;\mathrm{lr}(s)=\frac{1}{2}[(s_{-}^{T}\Sigma_{-}^{-1}s_{-})-(s_{+}^{T}\Sigma_{+}^{-1}s_{+})]+c.$$ (3)

For the variables, $s_{+}=s-\mu_{+}$ and $s_{-}=s-\mu_{-}$, and $c=\log|\frac{\Sigma_{-}}{\Sigma_{+}}|$, with $\mu_{+}$ and $\mu_{-}$ as the mean vectors and $\Sigma_{-}$ and $\Sigma_{+}$ as the covariance matrices for the incorrect and correct predictions, respectively. As shown in the calibrated predictions block in Fig. 2, a new confidence score for each prediction is obtained by mapping the input to the calibration map g. The ECEs of the prediction results before and after calibration are calculated to test the effect of calibration on model uncertainty.

## 3 Results

### 3.1 Experimental Setup

For model development, we use 943 OCT images from 14 OCT specimen segments for a threefold cross validation. The OCT images were acquired from specimens that contain calcification regions, which include essential information for CAD treatments. Within each OCT volume, B-scans were sampled at an interval of 20 B-scans. Each B-scan has a size of 1024 × 1500 pixels, corresponding to a space of $1.98\times 3\;{\mathrm{mm}}^{2}$. In the confidence calibration stage, 60% predictions are used to fit the calibration model, with the remaining 40% predictions to be tested. The ground-truth annotations used for training and testing were made under the guidance of the pathologists.

The YOLO was built in Python 3.8, PyTorch 1.10, CUDA 11.1, and NVIDIA RTX 6000, and a pretrained weight^53^ was used in this work, with a batch size of 8 and a learning rate of 0.001 using the Adam optimizer with a weight decay of 0.01. Two other popular object detection deep learning models were implemented to show the effectiveness of the calibration. A single-shot multibox detector (SSD)^54^ and faster region-based convolutional neural networks (Faster RCNN)^55^ were built in Python 3.8, PyTorch 1.10, CUDA 11.1, and NVIDIA RTX 6000. The training process of the SSD was started by loading the pretrained weight,^56^ with a batch size of 8 and a learning rate of 0.001 using the stochastic gradient descent optimizer with a momentum of 0.9. Faster RCNN used a pretrained weight^57^ and was trained with a batch size of 8 and a learning rate of 0.0001 using the Adam optimizer with a weight decay of 0.001.

### 3.2 Object Detection

To evaluate the performance of calcification detection, three metrics, precision, recall, and f1-score, are calculated, as given in the following eqautions:

$$\text{precision}=\frac{\mathrm{TP}}{\mathrm{TP}+\mathrm{FP}},$$ (4)

$$\text{recall}=\frac{\mathrm{TP}}{\mathrm{TP}+\mathrm{FN}},$$ (5)

$$f1\text{-score}=\frac{2\times \text{precision}\times \text{recall}}{\text{precision}+\text{recall}},$$ (6)

where the true positive (TP) means the model correctly predicts the region with calcification, the false negative (FN) is the wrong prediction for the region that has calcification, and the false positive (FP) is the wrong prediction for the region with no calcification. Precision indicates the number of correct predictions among all detections. Recall measures the fraction of correct predictions among ground truths. The f1-score is a measure of overall model performance determined by combining precision and recall.

**Fig. 3:**
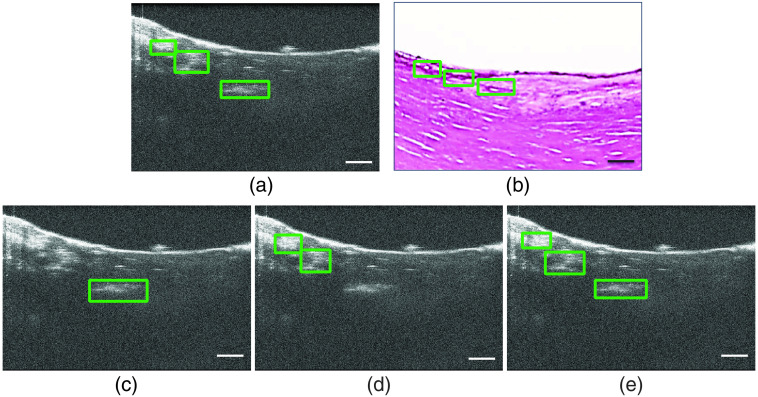
Example of (a) ground-truth label, (b) corresponding histology, and object detection results from (c) Faster RCNN, (d) SSD, and (e) YOLO. Scale bar: 500  μm.

**Fig. 4:**
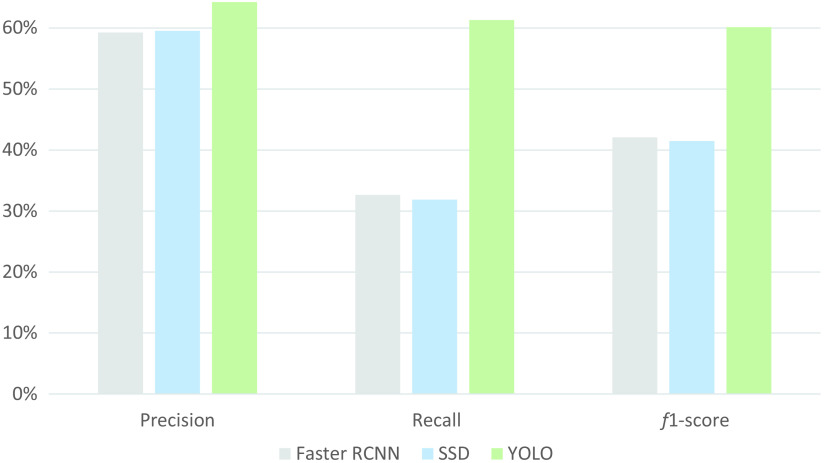
Object detection results of deep learning models with a threshold of 0.4 in precision, recall, and f-1 score. The gray bars are the results of Faster RCNN, the blue bars are the results of the SSD, and the green bars are the results of YOLO.

Qualitatively, in Fig. 3, YOLO predicts all calcification in this coronary OCT image. The SSD and Faster RCNN fail to detect the calcification region in relatively lower contrast. The low recall of the SSD and Faster RCNN in Fig. 4 reveals higher FN predictions, which agrees with the observation in Fig. 3.

In addition, as shown in Table 1, the processing speed of YOLO is 140 frames per second (fps), showing that YOLO has great capability for real-time detection, which is especially desirable in the circumstance of processing a large volume of OCT images. The runtimes of the SSD and Faster RCNN are 68 and 35 fps, respectively. The runtime of OCT segmentation of DeepRetina^58^ and CNN-S^59^ is ∼5  fps, which indicates a larger computational burden than detection.

**Table 1:** Runtime in fps for deep learning models detecting calcification in coronary OCT images in this work.

	Faster RCNN	SSD	YOLO
Runtime (fps)	35	68	140

### 3.3 Uncertainty Measurement and Confidence Calibration

**Fig. 5:**
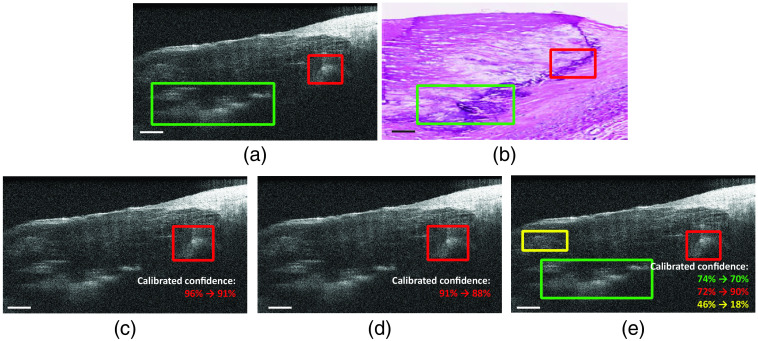
Example result: (a) ground-truth label, (b) corresponding histology, and confidence calibration results of (c) Faster RCNN, (d) SSD, and (e) YOLO. Scale bar: 500  μm.

We evaluate the effectiveness of the calibration of predictions for the deep learning models. In Fig. 5, the adjustment of confidence scores is observed in the calibrated predictions. In Figs. 5(c) and 5(d), the predictions from Faster RCNN and the SSD in the red box show that the confidence score is slightly adjusted. In Fig. 5(e), the overconfident predictions shown in the yellow box reduces the confidence score from 46% to 18% after calibration, whereas the other confidence scores of predicted boxes are slightly adjusted.

For quantitative evaluation, we use the ECE to measure the uncertainty using the mean value of all test targets. In Table 2, before calibration, YOLO has a lower level of uncertainty in ECE, indicating that YOLO produces more reliable predictions. For the three deep learning models, the calibration errors are lowered to the same level around ∼0.14 after calibration, which shows the effectiveness of the calibration process that helps rectify the overconfident predictions.

**Table 2:** Uncertainty measurements of confidence calibration in ECE. The rows of before/after calibration shows the changes in ECE during the calibration.

	Faster RCNN	SSD	YOLO
ECE	0.429	0.731	0.233
Calibrated ECE	0.151	0.134	0.146
Before/after calibration	0.278	0.585	0.099

## 4 Discussion and Conclusion

In this work, we reported calcification detection in coronary OCT images using deep learning models with uncertainty measurements and confidence calibration to reduce the bias in deep learning models. Although tissue detection and segmentation in OCT images have been studied, to our best knowledge, this work is the first to implement uncertainty measurement and confidence calibration for deep learning-based calcification detection in coronary OCT images. We investigated the calcification detection performance of deep learning object detection models and evaluated the reliability of predictions by detection accuracy and uncertainty measures. With an exceptional runtime of 140 fps, YOLO had the potential to become the real-time detector for predicting calcification in coronary OCT images. This work also implemented confidence calibration by integrating the bounding box information with the confidence score. The quantitative and qualitative results showed the effectiveness of the calibration, indicating its practical value in safe-critical and risk-sensitive applications, for example, the calcification detection in coronary OCT images during PCI.

In the future, we will implement other calibration methods on the predicted confidence score and seek to ensemble multiple models to produce more robust and reliable predictions for calcification detection in OCT images. Furthermore, by providing additional information critical to diagnosis, the calibrated confidence and uncertainty measures can be used in future clinical practice.

## Data Availability

The datasets generated and analyzed in this work are available from the corresponding author upon reasonable request.
